# Supplementary material for: Embedding biocatalysts in a redox polymer enhances the performance of dye-sensitized photocathodes in bias-free photoelectrochemical water splitting
Source: Nat Commun. 2024 Apr 13;15:3202. doi: 10.1038/s41467-024-47517-9 (PMC11016092; doi:10.1038/s41467-024-47517-9)
Supplement: Supplementary file 1 — Supplementary Information [file 41467_2024_47517_MOESM1_ESM.pdf]

Supplementary information

Embedding Biocatalysts in a Redox Polymer Enhances the Performance of Dye-Sensitized Photocathodes in Bias-free Photoelectrochemical Water Splitting

Fangwen Cheng,[1] Olha Pavliuk,[2] Steffen Hardt,[3] Leigh Anna Hunt,[1] Bin Cai,[1] Tomas Kubart,[4] Leif Hammarström,[1] Nicolas Plumeré*,[5], Gustav Berggren*,[2], Haining Tian*,[1]

[1] Department of Chemistry—Ångström laboratory, Physical Chemistry, Uppsala University, Box 521, 75120 Uppsala, Sweden

[2] Department of Chemistry—Ångström laboratory, Molecular Biomimetics, Uppsala University, Box 523, 75120 Uppsala, Sweden

[3] Institute of Energy and Climate Research, Fundamental Electrochemistry (IEK-9), Forschungszentrum Jülich GmbH, Wilhelm-Johnen-Straße, 52425 Jülich, Germany.

[4] Department of Electrical Engineering, Solid-State Electronics, Uppsala University, Box 65, 75103 Uppsala, Sweden

[5] TUM Campus Straubing for Biotechnology and Sustainability, Technical University of Munich, Uferstrasse 53, 94315 Straubing, Germany

Contents

Supplementary Method S2

Preparation of *Cr*HydA1 .... S2

Synthesis of viologen-modified polymer PolyV S2

Results S3

Redox potential measurement for the redox polymer (PolyV) S3

Cross-sectional SEM images S3

Optimization for the fabrication of the dye-sensitized photocathode S4

Incident photon-to-current efficiency and absorbed photon-to-current efficiency measurement S7

PEC performance of the photocathode in varied pH conditions S8

Characterizations for the stability of PB6 .... S8

Time-correlated single photon counting measurements S9

Spectroelectrochemistry spectra of PolyV S10

Femtosecond transient absorption (fsTA) measurements S11

Absorbance and stability of tandem devices S13

Summary for performance of NiO-based dye-sensitized photocathodes S14

References S15

Supplementary Method

Preparation of *Cr*HydA1.

Overexpression: Escherichia coli BL21(DE3) cells containing the *Cr*HydA1 plasmid were cultivated in M9 medium with ampicillin under aerobic conditions until reaching an optical density (OD_{600} = 0.6) of 0.6-0.8. The induction of protein overproduction was achieved using 1 mM IPTG and was maintained at 20 °C for 16-18 hours with continuous aeration. Additionally, 100 µM $FeSO_4$ was introduced into the medium upon induction.

Reconstitution: The in vitro reconstitution protocol for [4Fe4S] clusters in *Cr*HydA1 closely followed the previously outlined procedure with slight modifications.[1] In a standard reaction, HydA1 was subjected to incubation with L-cysteine (6 molar equivalents), Mohr's salt (6 molar equivalents), dithiothreitol (DTT, 10 molar equivalents), and cysteine desulfurase CsdA (0.02 molar equivalents) within a Tris-HCl buffer (100 mM Tris-HCl, 150 mM NaCl, pH 8.0). When the absorbance at approximately 410 nm reached a stable level, the reaction mixture underwent filtration using a NAP25 desalting column (GE Healthcare). The protein samples were promptly frozen under anaerobic conditions using liquid nitrogen outside of a glovebox and stored at a temperature of –80 °C for subsequent use.

In a standardized procedure,[1] HydA1 underwent treatment with $[Fe_2(adt)(CO)_4(CN)_2]^{2-}$ ($[2Fe]^{adt}$, at 6 molar equivalents) and sodium dithionite (NaDT, at 20 molar equivalents) at room temperature for a duration of 1 to 2 hours under anaerobic conditions. The reaction was halted by passing the solution through a NAP25 desalting column that had been pre-equilibrated with 10 mM Tris-HCl at pH 8.0. This process enabled a clear separation between the unreacted $[2Fe]^{adt}$ and $[2Fe]^{adt}$-HydA1.[2] The elution fractions containing $[2Fe]^{adt}$-HydA1 were concentrated, divided into aliquots, and promptly frozen in a manner similar to the preceding step.

Synthesis of viologen-modified polymer PolyV.

The redox polymer (PolyV) was synthesized following the reported method.[3] 1 g of Polyvinyl alcohol (PVA, 205,000 g mol-1) was dissolved in water (19 mL) and precipitated from a 1:1 solution of acetone and methanol (40 mL). The purified PVA (20 mg) was dried and dissolved in dry DMSO (4 mL). A solution of NCO-V2 (400 mg, 0.072 mmol) in DMSO (1 mL) was added followed by dibutyltin dilaurate (50 µL, 53.3 mg, 84.5 µmol). The detailed synthesis method for NCO-V2 was shown in Reference.[3] The solution was stirred for 24 h at room temperature under an Ar atmosphere. The crude product solution was diluted with distilled H_2O until the DMSO amount did not exceed 5% vol. The resulting polymer solution was purified via Vivaspin systems (5 kDa).

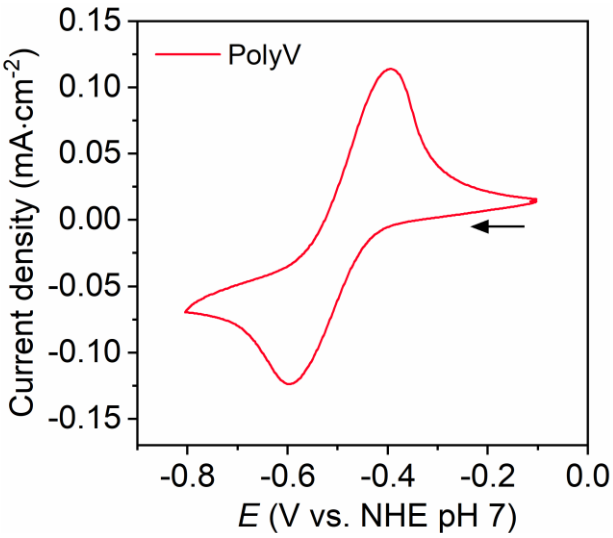

Supplementary Fig. 1. Cyclic voltammograms of PolyV on FTO. The CV was measured in 5 mM Tris buffer (pH 7) with a scan rate of 10 $mV{\cdot}s^{-1}$. Reduction of PolyV is reversible and the reduced potential for PolyV is determined to be –0.59 V vs. NHE at pH 7.

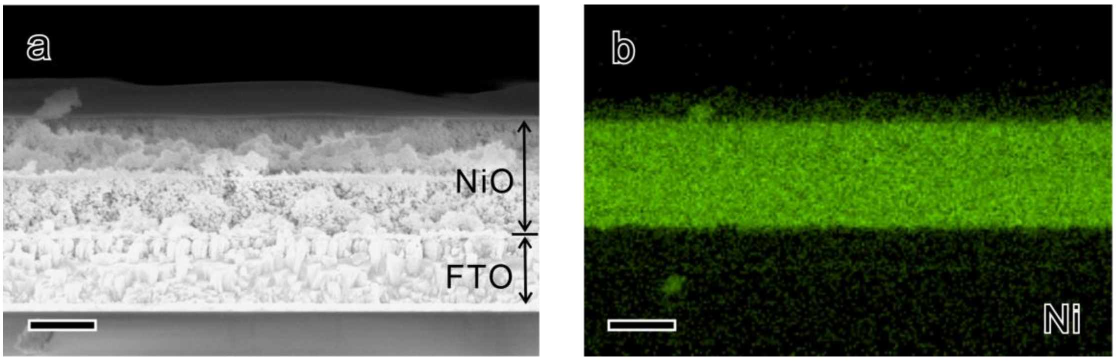

Supplementary Fig. 2. Cross-sectional SEM images. (a) Cross-sectional SEM images for NiO|PB6|PolyV|H_2ase photocathode. EDX images for (b) Ni. The scale bar is 500 nm.

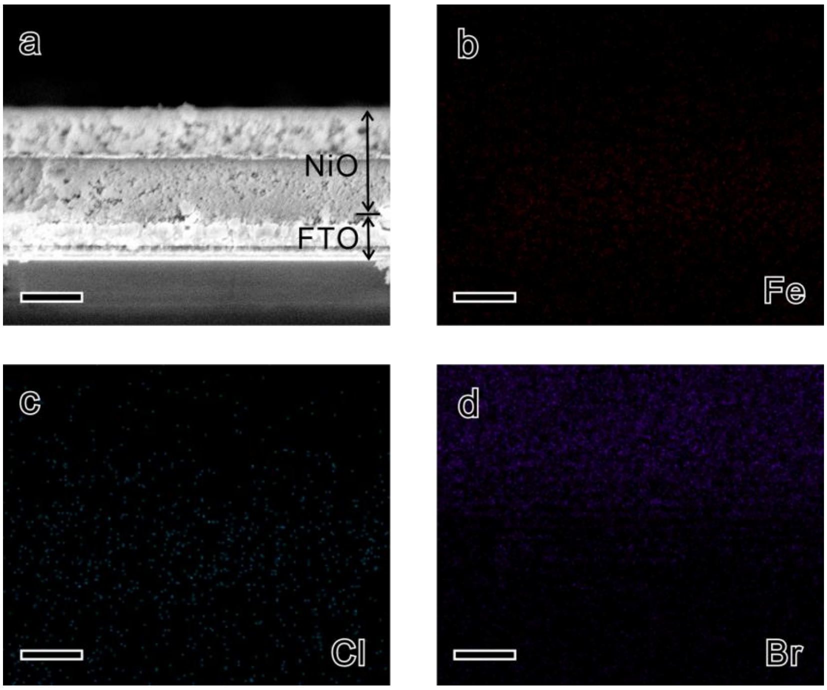

Supplementary Fig. 3. Cross-sectional SEM images. (a) Cross-sectional SEM images for NiO|PB6 photocathode. EDX images for (b) Fe, (c) Cl, and (d) Br. The scale bar is 500 nm.

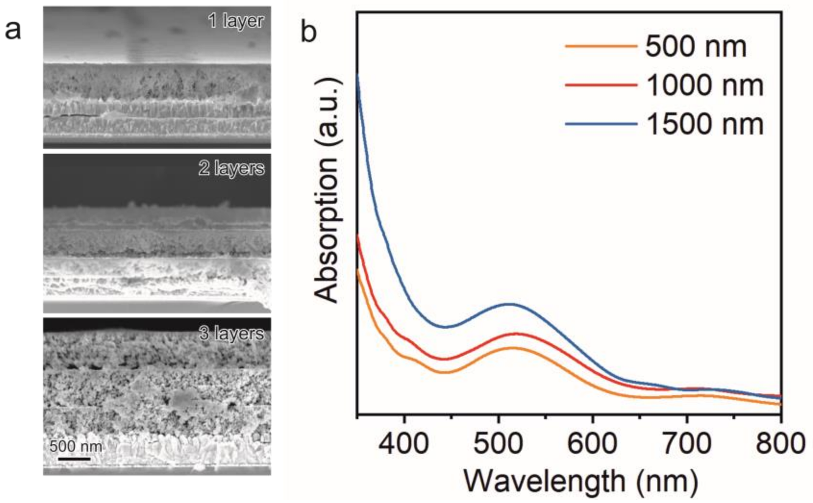

Supplementary Fig. 4. Optimization for the fabrication of the dye-sensitized photocathode. (a) Cross-sectional SEM images to determine the thickness of nanoporous NiO layer. (b) UV-vis absorption spectra of PB6-sensitized NiO films with different thicknesses.

NiO layers with different thicknesses were obtained by blade-coating/annealing for varied times. As shown in the cross-sectional SEM images, the thicknesses of single, double, and triple nanoporous NiO layers are 500, 1000, and 1500 nm.

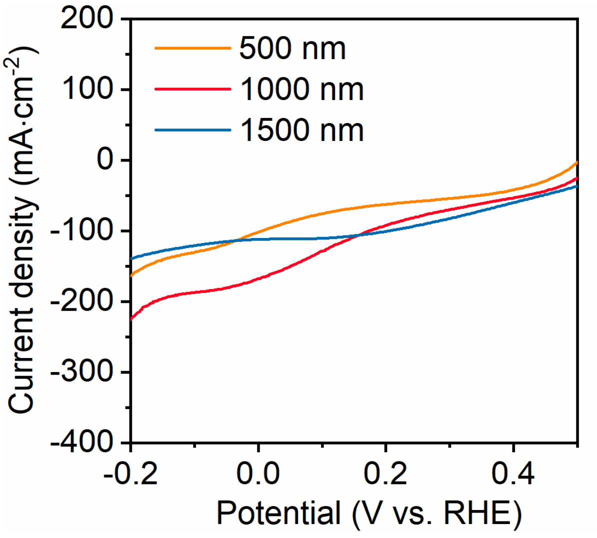

Supplementary Fig. 5. Linear sweep voltammetry (LSV) plots. NiO|PB6|PV|H_2ase photocathodes with various thicknesses of NiO (scan rate: 10 mV·s^{-1}) were conducted in 5mM Tris buffer.

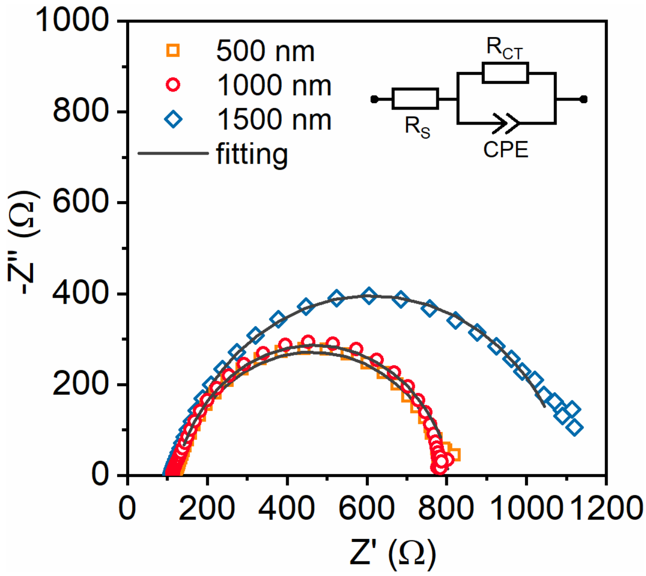

Supplementary Fig 6. Electrochemical impedance spectroscopy. Nyquist plots (scatter) and fittings (lines) for NiO|PB6|PV|H_2ase photocathode with various thicknesses of mesoporous NiO, conducted under illumination at a potential of –0.2 V vs. RHE in 5mM Tris buffer.

Electrochemical impedance spectroscopy provides information on the charge transfer transport kinetics on the electrode. The Nyquist plots can fit into a simple $R_S(R_{CT}/CPE)$ model, where R_S is the ohmic series resistance, R_{CT} is the charge transfer resistance associated with the total resistance of charge transport and recombination, and CPE is the constant-phase element representing the Helmholtz capacitance on uneven surfaces. According to the fitting results, R_S values of the photoelectrodes with different nanoporous NiO were consistent at around 110 Ω.

R_{CT} values were 687 and 691 Ω for the electrode with 500-nm and 1000-nm NiO, respectively, while it raised to 1036 Ω for the sample with a thicker NiO layer, showing more charge recombination behavior.

The NiO thickness significantly affected the photocurrent due to varied light absorption and charge transport resistance. The photocathode reached the highest photocurrent with 1000 nm mesoporous NiO, exhibiting optimal light absorption and resistance.

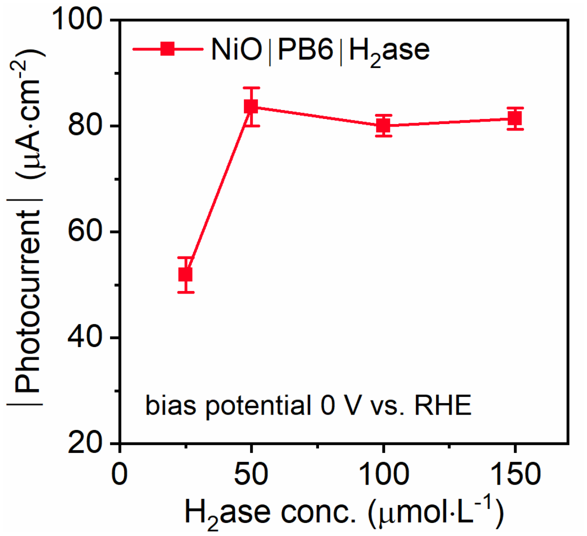

Supplementary Fig. 7. Optimization of H_2ase amounts. The photocurrent density value at 0 V vs. RHE for NiO|PB6|H_2ase with different H_2ase amounts. The concentration of the H_2ase solution varied and the deposited volume of the H_2ase solution was kept the same as 5 μL. The photocurrent values were extracted from LSV scans in 5mM Tris buffer.

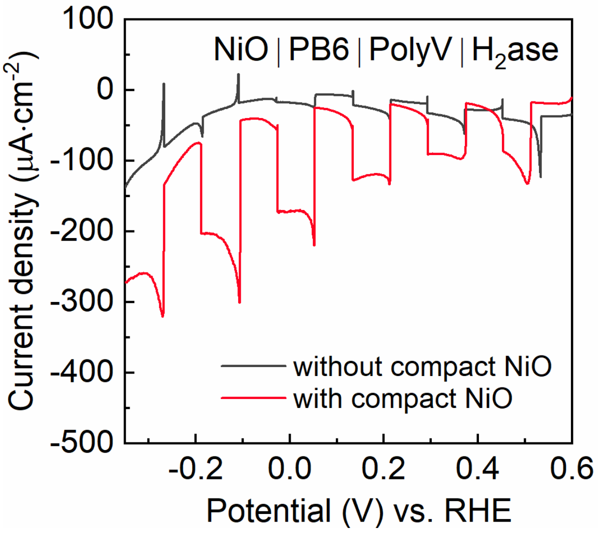

Supplementary Fig. 8. LSV plots. NiO|PB6|PolyV|H_2ase photocathode with or without compact NiO as the blocking layer. (scan rate: 10 mV·s^{-1}, 5 mM Tris buffer)

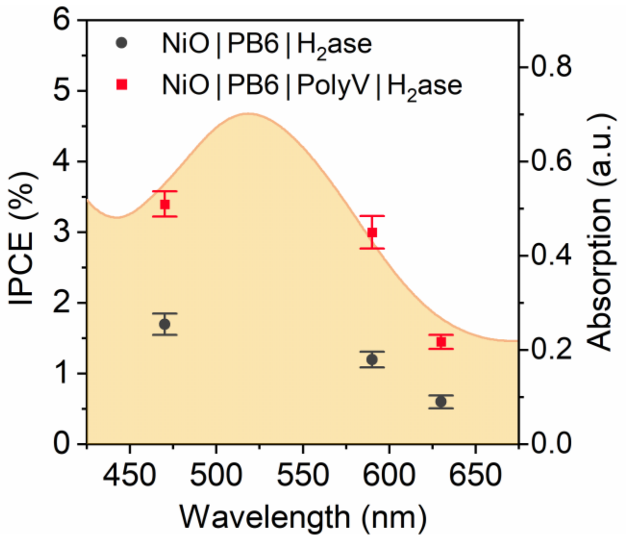

Supplementary Fig. 9. Incident photon-to-current efficiency (IPCE) and absorbed photon-to-current efficiency (APCE) measurement. IPCE at 470 nm, 590 nm and 630 nm for NiO|PB6|H_2ase and NiO|PB6|PolyV|H_2ase photocathode and absorption spectrum of PB6. IPCE is calculated with the photocurrent at 0 V vs. RHE obtained from LSV (scan rate: 10 mV·s^{-1}, 5 mM Tris buffer). IPCE for NiO|PB6|H_2ase is 1.7%, 1.2%, and 0.6% at 470 nm, 590 nm, and 630 nm, respectively. IPCE for NiO|PB6|PolyV|H_2ase is 3.4%, 3%, and 1.45% at 470 nm, 590 nm, and 630 nm, respectively. APCE for NiO|PB6|H_2ase is 2.4%, 1.9%, and 1.3% at 470 nm, 590 nm, and 630 nm, respectively. APCE for NiO|PB6|PolyV|H_2ase is 4.7%, 4.7%, and 3.2% at 470 nm, 590 nm, and 630 nm, respectively.

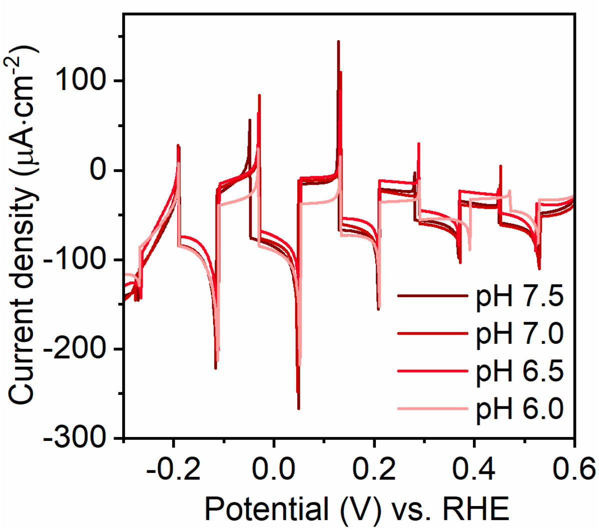

Supplementary Fig. 10. PEC performance of the photocathode in varied pH conditions. LSV measurements of NiO|PB6|PolyV|H_2ase in the electrolyte with pH varied from 6.0 to 7.5 under chopped light. The measurements used another batch of H_2ase with the activity of ~ 90 μmol H_2·(mg protein)$^{-1}$·min^{-1}.

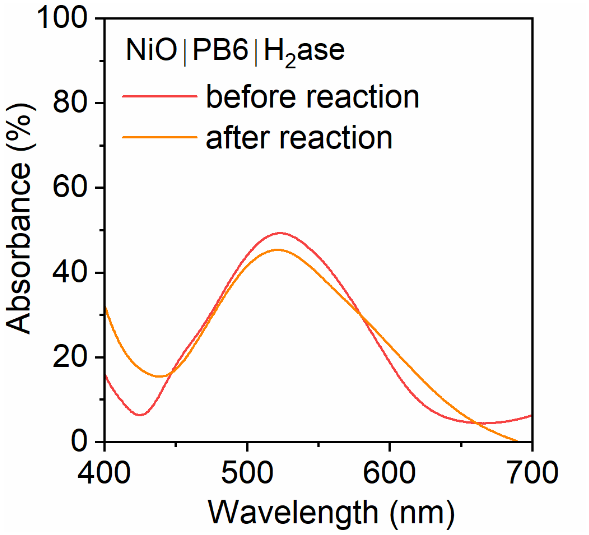

Supplementary Fig. 11. Characterizations for the stability of PB6. Absorption of PB6 on the photoelectrode before and after a 5-h reaction. The comparison of PB6 absorption peak at around 520 nm shows that PB6 dye remains stable after 5-h reaction.

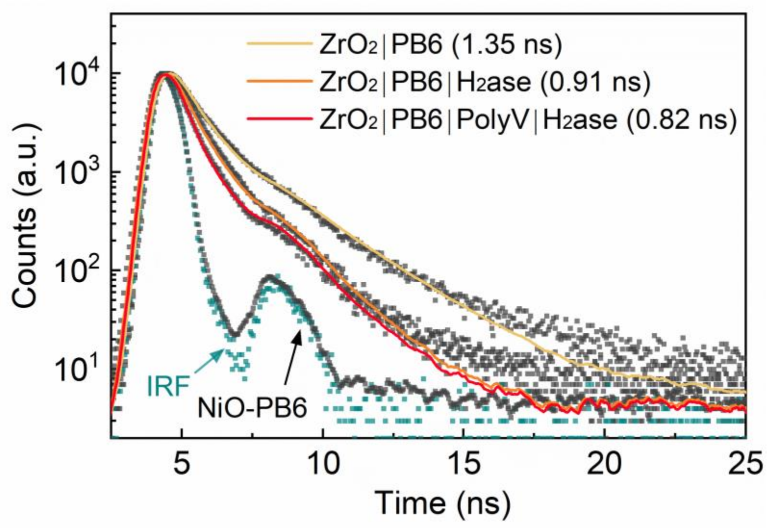

Supplementary Fig. 12. Time-correlated single photon counting (TCSPC) measurements. TCSPC curves for the PL decay following 470 nm pulsed laser excitation. The samples with PB6 adsorbed onto ZrO_2 reveal the electron extraction rate from excited PB6 and the sample with PB6 on NiO reveals the electron injection rate from NiO to excited PB6. The NiO|PB6 sample exhibited ultrafast photoluminescence decay on a time scale shorter than that of the instrument response function (IRF). Thus, femtosecond transient spectroscopy is requisite to track carrier dynamics of the excited state and the reduced state of PB6 adsorbed on NiO.

Supplementary Table 1. Fit parameters extracted from TCSPC measurements.

	τ_1 (ns)	τ_2 (ns)	$\tau_{average}$ (ns)
ZrO_2|PB6	0.65 (58.3%)	2.32 (41.7%)	1.35
ZrO_2|PB6|H_2ase	0.54 (63.9%)	1.57 (36.1%)	0.91
ZrO_2|PB6|PolyV|H_2ase	0.39 (64.1%)	1.61 (35.9%)	0.82

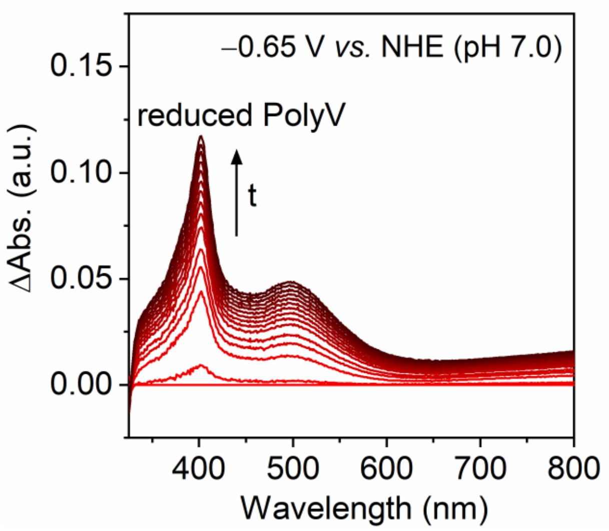

Supplementary Fig. 13. Spectroelectrochemistry spectra of PolyV. Time-resolved spectroelectrochemical scans of PolyV, showing the differential absorption recorded with an applied potential of –0.65 V *vs.* NHE in buffer solution with pH 7. The reduced PolyV exhibited two absorption peaks at around 400 nm and 500 nm. In fs-TAS (Fig. 4b), the peak shown at 400 nm proved the formation of reduced PolyV, but the peak at 500 nm was not shown in fs-TAS due to overlap with the ground state bleach of PB6.

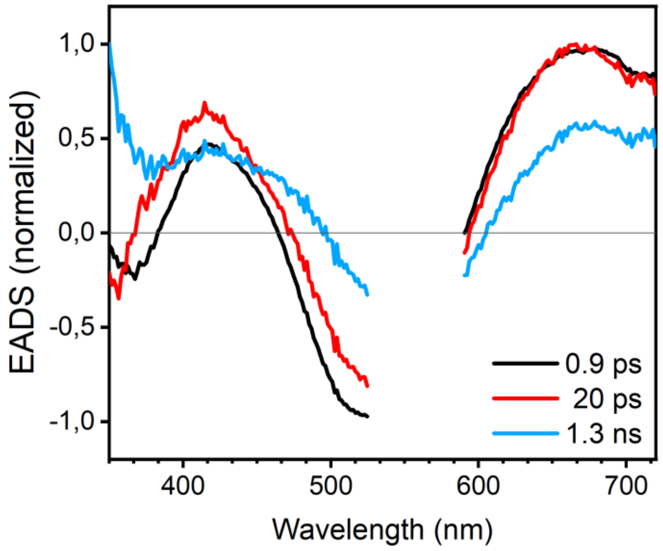

Supplementary Fig. 14. Femtosecond transient absorption (fsTA) measurements. Evolution-associated decay spectra (EADS) for NiO|PB6.

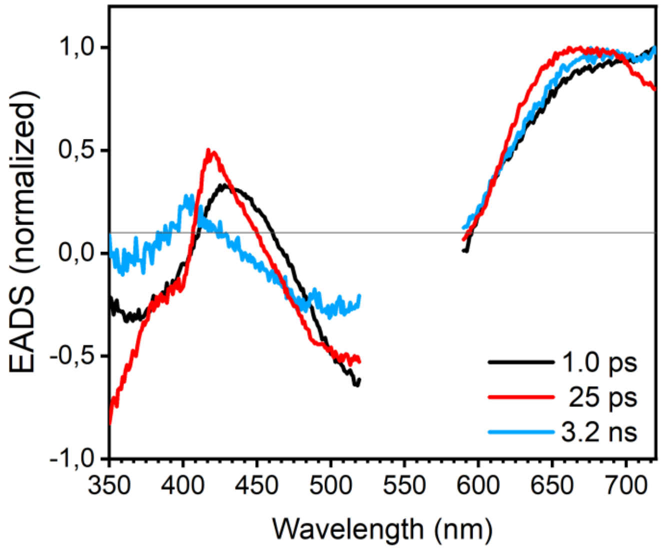

Supplementary Fig. 15. Femtosecond transient absorption (fsTA) measurements. Evolution-associated decay spectra (EADS) for NiO|PB6|PolyV.

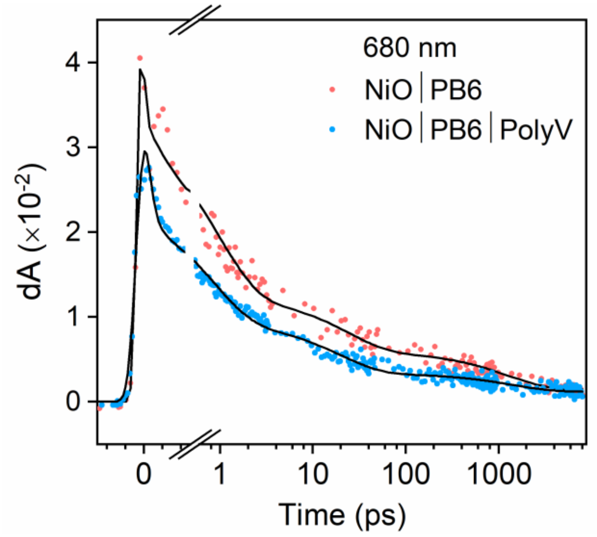

Supplementary Fig. 16. Decay of reduced PB6. Single wavelength kinetics at 680 nm for NiO|PB6 and NiO|PB6|PolyV films.

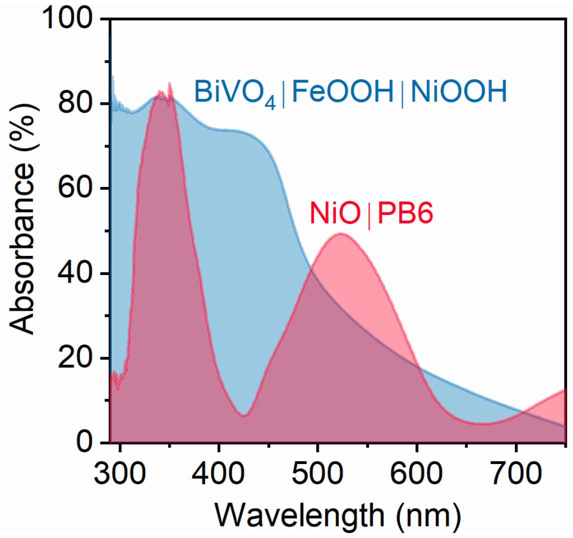

Supplementary Fig. 17. Absorbance of tandem devices. The absorbance of modified $BiVO_4$ and NiO|PB6 electrode.

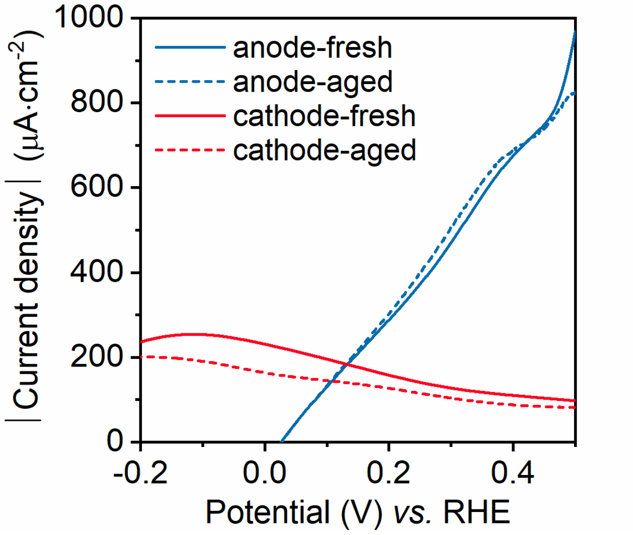

Supplementary Fig. 18. Stability of tandem devices. LSV scans for the $BiVO_4$ photoanode and the dye-sensitized photocathode before and after 10 h of operation, showing that $BiVO_4$ photoanode is stable. The decay in the photocurrent of tandem cells is ascribed to the deactivation of photocathode.

Summary for performance of NiO-based dye-sensitized photocathodes

Supplementary Table 2. Performance of NiO-based dye-sensitized photocathodes for H_2 generation.

Sensitizer	Catalyst	Applied potential (electrolyte pH)	Photocurrent density (illumination time)	Faradaic efficiency	Ref.
P1	$CodmgBF_2$	–0.4 V *vs* Ag/AgCl (pH 7.0)	20 μA·cm^{-2} (300 s)	–	4
PMI-6T-TPA	–	0 V *vs* Ag/AgCl (pH 7.0)	3.9 μA·cm^{-2} (4 h)	97%	5
O22	$CodmgBF_2$	0.1 V *vs* NHE (pH 7.0)	9 μA·cm^{-2} (1 h)	45%	6
RuP	Co(III) complex	–0.4 V *vs* Ag/AgCl (pH 7.0)	13 μA·cm^{-2} (150 s)	–	7
RuP	Rh(III) complex	−0.45 V *vs* $Ag/AgNO_3$	12.5 μA·cm^{-2} (4 h)	85%	8
P1	$CodmgBF_2$	−0.2 V *vs* Ag/AgCl (pH 7.0)	20 μA·cm^{-2} (90 min)	68%	9
BH4	Mo_3S_4 cluster	−0.17 V *vs* NHE (pH 0)	183 μA·cm^{-2} (16.6 h)	49%	10
RuP3	NiP	0.3 V *vs* RHE (pH 3.0)	6.40 μA·cm^{-2} (100 s)	10%	11
PMI-6T-TPA	Pt	0.059 V *vs* RHE (pH 1.0)	30 μA·cm^{-2} (12 h)	~100%	12
Covalent dye-catalyst assembly (Organic dye and Co complex)		0.14 V *vs* RHE (pH 5.5)	15 μA·cm^{-2} (600 s)	8−10%	13
PMI	NiL_2	–0.4 V *vs* Ag/AgCl (pH 0.7)	175 μA·cm^{-2} (60 s)	98%	14
PB-1	$Co\text{-}N_3$	0 V *vs* Ag/AgCl (pH 6.8)	10 μA·cm^{-2} (600 s)	–	15
Covalent dye-catalyst assembly (Ru^{II}-Zr-Ni^{II})		−0.25 V *vs* NHE (pH 5.0)	40 μA·cm^{-2} (40 s)	~90%	16
RBG-174	Co_{C11P}	0.14 V *vs* RHE (pH 5.5)	8 μA·cm^{-2} (5 min)	9.3%	17
$ZnPRu^{2+}$	$NiMo_{0.05}S_x$	−0.15 V *vs* NHE (pH 4.5)	95 μA·cm^{-2} (2 min)	78.5%	18
NPP	$CodmgBF_2$	0 V *vs* Ag/AgCl (pH 7.0)	13 μA·cm^{-2} (200 s)	–	19
Covalent dye-catalyst assembly (Ru^{II}-Zr-Co^{II})		–0.4 V *vs* Ag/AgCl (pH 4.5)	1 μA·cm^{-2} (4.5 h)	27%	20
T1	$CodmgBF_2$	−0.4 V *vs* Ag/AgCl (pH 4.5)	6 μA·cm^{-2} (500 s)	3%	21
RBG-174	CoHEC	0.14 V *vs* RHE (pH 5.5)	6 μA·cm^{-2} (2 h)	70%	22

PB6	Pt	0.05 V *vs* RHE (pH 5.0)	80 $\mu A\cdot cm^{-2}$ (2 h)	~100%	23
PB6	Co complex	0.1 V *vs* NHE (pH 5.0)	16 $\mu A\cdot cm^{-2}$ (100 s)	–	24
Sil-PB6	Co complex	0.1 V *vs* NHE (pH 5.0)	4 $\mu A\cdot cm^{-2}$ (1 h)	–	25
Covalent dye-catalyst assembly (T2R-Cat1)		−0.4 V *vs* Ag/AgCl (pH 5.5)	6.3 $\mu A\cdot cm^{-2}$ (20 min)	66%	26
Covalent dye-catalyst assembly (ZnP-Co)		−0.4 V *vs* Ag/AgCl (pH 5.5)	2.5 $\mu A\cdot cm^{-2}$ (2 h)	8%	27
Covalent dye-catalyst assembly (RuP_4-Co)		0.14 V *vs* RHE (pH 7.0)	< 5 $\mu A\cdot cm^{-2}$ (2 h)	26%	28
QAP-C8	Co2	-0.1 V vs NHE (pH 7.0)	12 $\mu A\cdot cm^{-2}$ (100 s)	–	29
PCA (AuNPs)	Co2	−0.1 V *vs* Ag/AgCl (pH 5.0)	10 $\mu A\cdot cm^{-2}$ (15 min)	10%	30
Covalent dye-catalyst assembly (TAPy-Co2)		−0.2 V *vs* Ag/AgCl (pH 5.0)	63$\mu A\cdot cm^{-2}$ (6 h)	9.1%	31

Supplementary References

1. Lorenzi, M. *et al.* Investigating the role of the strong field ligands in [FeFe] hydrogenase: spectroscopic and functional characterization of a semi-synthetic mono-cyanide active site. *Chem. Sci.* **13**, 11058-11064 (2022).
2. Zamader, A. *et al.* Synthetic styrene-based bioinspired model of the [FeFe]-hydrogenase active site for electrocatalytic hydrogen evolution. *Sustainable Energy & Fuels* **7**, 4967-4976 (2023).
3. Hardt, S. *et al.* Reversible H_2 Oxidation and Evolution by Hydrogenase Embedded in a Redox Polymer Film. *Nat. Catal.* **4**, 251-258 (2021).
4. Li, L. *et al.* Visible light driven hydrogen production from a photo-active cathode based on a molecular catalyst and organic dye-sensitized p-type nanostructured NiO. *Chem. Commun.* **48**, 988-990 (2012).
5. Tong, L. *et al.* Sustained solar hydrogen generation using a dye-sensitised NiO photocathode/$BiVO_4$ tandem photo-electrochemical device. *Energy Environ. Sci.* **5**, 9472 (2012).
6. Ji, Z., He, M., Huang, Z., Ozkan, U. & Wu, Y. Photostable p-type dye-sensitized photoelectrochemical cells for water reduction. *J. Am. Chem. Soc.* **135**, 11696-11699 (2013).
7. Fan, K. *et al.* Pt-free tandem molecular photoelectrochemical cells for water splitting driven by visible light. *Phys. Chem. Chem. Phys.* **16**, 25234-25240 (2014).
8. Castillo, C. E. *et al.* Visible Light-Driven Electron Transfer from a Dye-Sensitized p-Type NiO Photocathode to a Molecular Catalyst in Solution: Toward NiO-Based Photoelectrochemical Devices for Solar Hydrogen Production. *J. Phys. Chem. C* **119**, 5806-5818 (2015).
9. Li, F. *et al.* Organic Dye-Sensitized Tandem Photoelectrochemical Cell for Light Driven Total Water Splitting. *J. Am. Chem. Soc.* **137**, 9153-9159 (2015).
10. Click, K. A., Beauchamp, D. R., Huang, Z., Chen, W. & Wu, Y. Membrane-Inspired Acidically Stable Dye-Sensitized Photocathode for Solar Fuel Production. *J. Am. Chem. Soc.* **138**, 1174-1179 (2016).

11. Gross, M. A., Creissen, C. E., Orchard, K. L. & Reisner, E. Photoelectrochemical hydrogen production in water using a layer-by-layer assembly of a Ru dye and Ni catalyst on NiO. *Chem. Sci.* **7**, 5537-5546 (2016).
12. Hoogeveen, D. A. *et al.* Photo-electrocatalytic hydrogen generation at dye-sensitised electrodes functionalised with a heterogeneous metal catalyst. *Electrochim. Acta* **219**, 773-780 (2016).
13. Kaeffer, N. *et al.* Covalent Design for Dye-Sensitized H_2-Evolving Photocathodes Based on a Cobalt Diimine-Dioxime Catalyst. *J. Am. Chem. Soc.* **138**, 12308-12311 (2016).
14. Kamire, R. J. *et al.* Photodriven hydrogen evolution by molecular catalysts using Al_2O_3-protected perylene-3,4-dicarboximide on NiO electrodes. *Chem. Sci.* **8**, 541-549 (2017).
15. Pati, P. B. *et al.* Insights into the Mechanism of a Covalently Linked Organic Dye-Cobaloxime Catalyst System for Dye-Sensitized Solar Fuel Devices. *ChemSusChem* **10**, 2480-2495 (2017).
16. Shan, B. *et al.* Modulating Hole Transport in Multilayered Photocathodes with Derivatized p-Type Nickel Oxide and Molecular Assemblies for Solar-Driven Water Splitting. *J Phys. Chem. Lett.* **8**, 4374-4379 (2017).
17. Kaeffer, N. *et al.* Insights into the mechanism and aging of a noble-metal free H_2-evolving dye-sensitized photocathode. *Chem. Sci.* **9**, 6721-6738 (2018).
18. Shan, B. *et al.* Direct photoactivation of a nickel-based, water-reduction photocathode by a highly conjugated supramolecular chromophore. *Energy Environ. Sci.* **11**, 447-455 (2018).
19. Zhang, S., Li, X., Yun, K., Yu, F. & Hua, J. Effects of Electrolytes on the Photocurrent of N-Annulated Perylene-Sensitized Photoelectrochemical Cells Based on NiO as Photocathode. *ChemElectroChem* **5**, 3198-3205 (2018).
20. Lyu, S. *et al.* H_2-Evolving Dye-Sensitized Photocathode Based on a Ruthenium–Diacetylide/Cobaloxime Supramolecular Assembly. *ACS Appl. Energy Mater.* **2**, 4971-4980 (2019).
21. Massin, J. *et al.* Investigating Light-Driven Hole Injection and Hydrogen Evolution Catalysis at Dye-Sensitized NiO Photocathodes: A Combined Experimental–Theoretical Study. *J. Phys. Chem. C* **123**, 17176-17184 (2019).
22. Windle, C. D. *et al.* Earth-Abundant Molecular Z-Scheme Photoelectrochemical Cell for Overall Water-Splitting. *J. Am. Chem. Soc.* **141**, 9593-9602 (2019).
23. Xu, B., Tian, L., Etman, A. S., Sun, J. & Tian, H. Solution-processed nanoporous NiO-dye-ZnO photocathodes: Toward efficient and stable solid-state p-type dye-sensitized solar cells and dye-sensitized photoelectrosynthesis cells. *Nano Energy* **55**, 59-64 (2019).
24. Materna, K. L. *et al.* Understanding the Performance of NiO Photocathodes with Alkyl-Derivatized Cobalt Catalysts and a Push-Pull Dye. *ACS Appl. Mater. Interfaces* **12**, 31372-31381 (2020).
25. Materna, K. L. *et al.* Using Surface Amide Couplings to Assemble Photocathodes for Solar Fuel Production Applications. *ACS Appl. Mater. Interfaces* **12**, 4501-4509 (2020).
26. Bold, S. *et al.* Spectroscopic Investigations Provide a Rationale for the Hydrogen-Evolving Activity of Dye-Sensitized Photocathodes Based on a Cobalt Tetraazamacrocyclic Catalyst. *ACS Catal.* **11**, 3662-3678 (2021).
27. Charisiadis, A. *et al.* Synthesis and Characterization of a Covalent Porphyrin-Cobalt Diimine-Dioxime Dyad for Photoelectrochemical H_2 Evolution. *Eur. J. Inorg. Chem.* **2021**, 1122-1129 (2021).
28. Giannoudis, E. *et al.* Hydrogen Production at a NiO Photocathode Based on a Ruthenium Dye-Cobalt Diimine Dioxime Catalyst Assembly: Insights from Advanced Spectroscopy and Post-operando Characterization. *ACS Appl. Mater. Interfaces* **13**, 49802-49815 (2021).
29. Shen, L. *et al.* Pure organic quinacridone dyes as dual sensitizers in tandem photoelectrochemical cells for unassisted total water splitting. *Chem. Commun.* **57**, 5634-5637 (2021).
30. Lalaoui, N. *et al.* Gold nanoparticle-based supramolecular approach for dye-sensitized H_2-evolving photocathodes. *Dalton Trans.* **51**, 15716-15724 (2022).
31. Tang, K., Shao, J. Y. & Zhong, Y. W. A multi-pyridine-anchored and -linked bilayer photocathode for water reduction. *Chem. Eur. J.*, e202302663 (2023).
